# Development and application of an indirect enzyme-linked immunosorbent assay using recombinant truncated Cap protein for the diagnosis of porcine circovirus-like virus P1

**DOI:** 10.1186/s12917-016-0641-8

**Published:** 2016-01-19

**Authors:** Li-bin Wen, Shi-fu Wen, Kong-wang He

**Affiliations:** Institute of Veterinary Medicine, Jiangsu Academy of Agricultural Sciences, Nanjing, 210014 China; Key Laboratory of Veterinary Biological Engineering and Technology, Ministry of Agriculture, Nanjing, 210014 China; National Center for Engineering Research of Veterinary Bio-products, Nanjing, 210014 China

**Keywords:** Porcine circovirus-like virus P1, ORF1 protein, ELISA, Swine farms

## Abstract

**Background:**

Porcine circovirus-like virus P1 is a newly discovered virus. To date, there has been no specific serological assay for use in the diagnosis of P1 infection.

**Results:**

Because P1 has high homology to porcine circovirus type 2 (PCV2) at the nucleotide level, the C-terminal portion of the capsid protein (amino acids 73–114), a discriminative antigen, was expressed in a prokaryotic expression system. The recombinant product (rctCap), composed of three identical repeated domains, was shown to be strongly immunoreactive to P1-specific serum. This assay was validated by comparison with an indirect immunofluorescence assay (IFA). The diagnostic sensitivity and specificity of the rctCap enzyme-linked immunosorbent assay (ELISA) developed in this study are 93.6 % and 98.3 %, respectively, compared with the results from IFAs on 450 sera samples from pigs.

**Conclusions:**

The indirect ELISA that we developed with rctCap, the recombinant capsid fragment containing the 217–342 nt repeat domain, was sensitive, specific, and suitable for the large-scale detection of P1 infections in swine.

## Background

Porcine circovirus (PCV) is a nonenveloped, single-stranded, DNA virus with a circular genome of approximately 1.7 kb, belonging to the family *Circoviridae*. Porcine circovirus type 1 (PCV1) was first isolated as a non-pathogenic contaminant virus in a porcine kidney cell line [1], whereas porcine circovirus type 2 (PCV2) is recognized as a causative agent of postweaning multisystemic wasting syndrome (PMWS) [2], a multifactorial swine disease first identified in Canada in 1991 [3]. PCV2 has since been reported in most pig-producing countries worldwide, including China [4–6]. The PCV2 genome contains three major open reading frames (ORFs): ORF1, ORF2, and ORF3 [7–9]. ORF1 encodes the 35.7-kDa Rep proteins, which are essential for viral DNA replication [10]; ORF2 encodes the 30-kDa structural capsid protein involved in the host immune response [11]; and the ORF3 protein is involved in PCV2-induced host cell apoptosis [12].

Porcine circovirus-like virus P1 was recently identified and has the smallest genome of all DNA viruses (648 nucleotides), which is highly homologous to that of PCV2. Like the PCVs, P1 has a single-stranded circular DNA genome. P1 is thought to be a recombinant virus formed between PCV2 and another virus [13], and it similarly contains three major ORFs: ORF1, ORF2, and ORF3. Recently, we reported that ORF1 of P1 encodes a major structural protein of approximately 12.5 kDa. This protein reacts strongly with serum from P1-infected swine, suggesting its possible utility in diagnostic assays [14]. A comparison of the deduced N-terminal amino acid sequence of P1 ORF1 revealed that it has a high homology with the N-terminal domain of PCV2 ORF2. However, they only share a low amino acid sequence homology in the C-terminal region because there has been a frameshift in the P1 ORF1.

Epidemiological studies have confirmed that the P1 virus is endemic on pig farms in China [15]. In vivo studies have shown that P1 infects pigs and the infected animals show the clinical signs of PMWS [16].

To determine the prevalence of P1 infection and to clarify how PMWS develops, specific tools for antibody detection are essential. Testing for P1 antibodies in sera is currently performed with indirect immunofluorescence assays (IFAs) or immunoperoxidase monolayer assays (IPMAs) [16]. However, these assays are very time-consuming, whereas enzyme-linked immunosorbent assays (ELISAs) can be automated and are more convenient. Notably, two antigenic epitopes of the P1 capsid (Cap) protein have been identified at amino acid residues 6–18 and 80–92. A peptide antibody derived from the immunorelevant 6–18 epitope was used in our previous investigations [17]. However, the resulting test was not specific for P1 because there is a slight amount of antigenic cross-reactivity between PCV2 and P1. A 2014 report demonstrated that a peptide antibody test using the 80–92 epitope was highly specific for P1. Therefore, we used this epitope to establish methods for the specific serological detection of P1 [17]. A recombinant C-terminally truncated capsid protein (rctCap) of P1, expressed in an *Escherichia coli* system, was used as an antigen to develop an indirect ELISA.

The objectives of the present study were to establish and validate a diagnostic indirect ELISA for the serological detection of anti–P1 serum antibodies.

## Methods

### Ethics statement

This study was approved by the Committee on the Ethics of Animal Experiments of the Institute of Veterinary Medicine, Jiangsu Academy of Agricultural Sciences (JAAS no 20100604) (Nanjing, China).

### Expression, purification, and identification of the rctCap protein

To construct a plasmid expressing rctCap, genomic DNA was obtained from P1-infected porcine kidney epithelial (PK15) cells and used as the template for polymerase chain reaction (PCR). To obtain the target gene, ORF1 without the N-terminal 216-nucleotides (nt), three pairs of primers (Table 1) were designed according to the published genome sequence of P1 strain JSNJ (GenBank: KJ612072).

**Table 1:** Sequences of primers used for the PCRs

Primers	Sequences (5′-3′)	Underlined restriction site
A1	TTATCCATGGGCCACCACCACCACCACCACACTACTCCTCCCGCCAT ACAAT	*Nco*I
A2	GCCGAATTCGCCAAAGCTGATTCTT	*Eco*RI
B1	ATGAATTCACTACTCCTCCCGCCATAC	*Eco*RI
B2	TTGGAGCTCGCCAAAGCTGATTCCT	*Sac*I
C1	ATAGAGCTCACTACTCCTCCCGCCATACAAT	*Sac*I
C2	CGAAGCTTATTAGCCAAAGCTGATTCCTTTTGTT	*Hin*d III

**Fig. 1:**
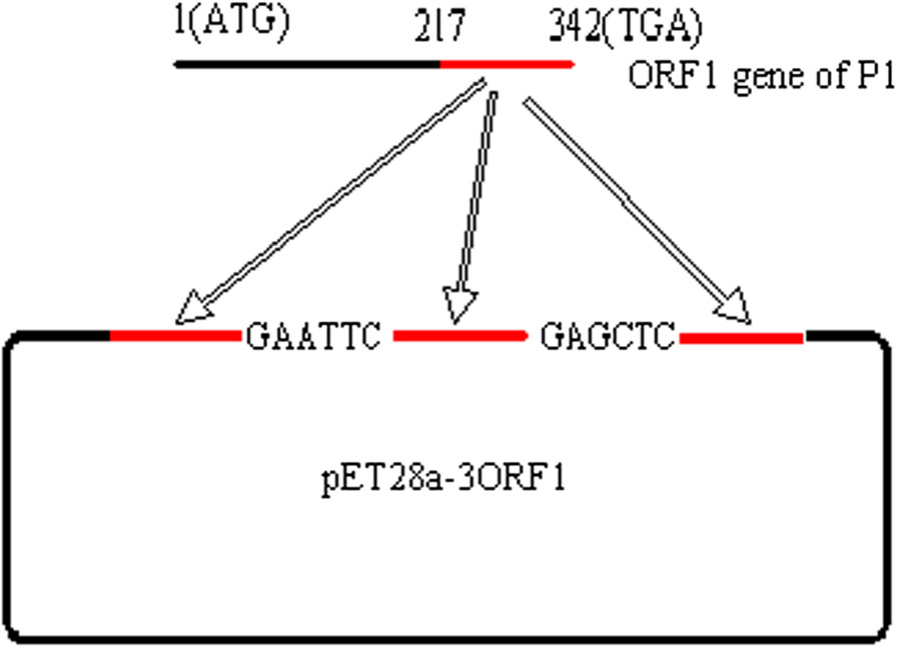
Construction of the rctCap expression plasmid. A schematic diagram showing the construction of the rctCap expression plasmid used in this study. The fragments of identical sequence are marked in red

The PCR product generated using the A1 and A2 primer pair was doubly digested with *Nco*I and *Eco*RI and cloned into the prokaryotic expression vector pET-28a (+) (Invitrogen, CA, USA). The resulting recombinant expression plasmid, pET28a-mono-ORF1 was ligated to the PCR product generated with the B1 and B2 primer pair and doubly digested with *Eco*RI and *Sac*I. The PCR product generated using the C1 and C2 primer pair was then digested with the *Sac*I and *Hin*dIII endonucleases and transferred into the above construct, resulting in the recombinant expression plasmid, pET28a-bi-ORF1. The pET28a-tri-ORF1 plasmid was then constructed similarly and used to transform competent *E. coli* DH5α cells (Fig. 1). Clones containing the recombinant plasmid were identified with restriction enzyme digestion and DNA sequencing (GenScript Corporation, China).

The rctCap plasmid has three tandem repeats, each encoding 42 amino acids. The three central repeats are identical except for a few linker amino acids.

To express the cloned gene, competent *E. coli* BL21-(DE3) cells (TransGen Biotech, China) were transformed with pET28a-tri-ORF1. Alternately, these cells were transformed with plasmid pET-28a (+) as a control. Single-positive clones of the transformed cells were grown in Luria-Bertani medium containing 80 μg/ml kanamycin at 37 °C (with shaking) to an optical density at 600 nm (OD_600_) of about 0.4–0.6. Protein expressin was induced in these cells by the addition of isopropyl β-d-1-thiogalactoside (IPTG) to a final concentration of 1 mM for 6–8 h at 37 °C. The bacteria were pelleted and resuspended in phosphate-buffered saline (PBS), and then lysed with sonication on ice. The sonicated cells were centrifuged at 10,000 × g for 30 min at 4 °C and the precipitate was resuspended and purified by Ni^2+^-NTA agarose (Qiagen, Germany), according to the manufacturer’s instructions. The eluates containing the rctCap protein were pooled and dialyzed against 0.01 M PBS (pH 7.2). The purified proteins were analyzed with sodium dodecyl sulfate–polyacrylamide gel electrophoresis (SDS-PAGE) and transferred electrophoretically to a nitrocellulose membrane. The membrane was blocked with blocking buffer (Tris-buffered saline containing 0.1 % Tween-20 and 2 % skim milk powder) overnight at 4 °C and then incubated with rabbit anti-P1 antibody (diluted 1:200) raised against the synthetic peptide CSPNPSPTTPVTSH. Horseradish peroxidase (HRP)-conjugated goat anti-rabbit IgG antibody (1:3000) (Boster Biological Technology, Wuhan, China) was used as the secondary antibody. Immunoreactivity was visualized with diaminobenzidine (Boster Biological Technology).

The concentration of the purified rctCap protein was determined with the Bradford method using bovine serum albumin (BSA, Sigma-Aldrich, St. Louis, MO, USA) as the standard, and the purified rctCap protein was stored at −70 °C until use in later assays.

### ELISA procedure

High-binding 96-well microtiter plates (Greiner BioOne, USA) were coated with 100 μl of rctCap protein in 0.05 M bicarbonate/carbonate buffer (pH 9.6) overnight at 4 °C. After three washes in PBS (pH 7.4) containing 0.05 % Tween 20 (PBS-T), the plates were blocked with 1 % BSA for 1 h at 37 °C. Serum samples were diluted in PBS (pH 7.4) containing 5 % dry milk, 100 μl of the diluted samples were added to the wells, and the plates were incubated for 1.5 h at 37 °C. The plates were then washed five times with PBS-T and incubated for 45 min at 37 °C with 100 μl of diluted HRP-conjugated goat anti-swine IgG antibody (KPL, Gaithersburg, MD). The plates were then washed four times, and the colorimetric reaction was developed with 50 μl of Tetramethylbenzidine Liquid Substrate (Sigma-Aldrich) for 10 min at 37 °C. The color reaction was stopped by the addition of 50 μl of 1 M H_2_SO_4_, and the OD_450_ was measured with a microplate reader (Bio-Tek Instruments, USA).

Based on the procedure described above, the optimal antigen concentration and serum dilution were determined with a checkerboard serum dilution analysis. In brief, the rctCap protein was serially diluted 2-fold from 5.24 μg/ml to 0.33 μg/ml. Additionally, P1-positive swine sera and P1 seronegative control swine sera were also subjected to a similar serial 2-fold dilution from 1:25−1:800 for use in the optimization of the rctCap ELISA. After the optimal antigen and antiserum dilutions were established, the HRP-labeled goat anti-swine IgG antibody was added to the plate at dilutions of 1:5000, 1:10,000, 1:20,000, and 1:40,000 to determine the optimal detection antibody dilution for this ELISA. The conditions that gave the highest positive control OD_450_/negative control OD_450_ (P/N) ratio and had an OD_450_ value close to 1.0 for positive serum were considered to be the optimal working conditions.

### Determination of the negative–positive cut-off

Nine hundred swine serum samples were collected sequentially from P1-free pigs that tested negative both for P1 antibodies via IFA and for P1 nucleic acids via PCR assays. These samples were used to set the negative–positive cut-off value for the rctCap ELISA. The mean S/P ratio (x) and standard deviation (SD) were calculated, and the cut-off value was calculated as x + (3 × SD).

### Evaluation of assay performance

To evalute the negative–positive cut-off value for this assay, 450 serum samples from farm pigs were tested in duplicate with the newly developed rctCap ELISA. The diagnostic accuracy of this ELISA was determined by calculating the diagnostic sensitivity and specificity of the test. IFA was used as the reference method to classify the samples as positive or negative. The sensitivity and specificity of the rctCap ELISA were determined as follows: sensitivity = (TP/[TP + FN]) × 100, and specificity = (TN/[TN + FP]) × 100, where TP, FN, TN and FP indicate the true-positive, false-negative, true-negative, and false-positive, respectively.

To detect the specificity of the rctCap ELISA, seropositive sera for PCV2, classical swine fever virus (CSFV), porcine parvovirus (PPV), porcine pseudorabies virus (PRV), or porcine reproductive and respiratory syndrome virus (PRRSV) were tested with the rctCap ELISA. Each sample was tested in triplicate, and the S/P ratios were calculated.

### Determination of assay reproducibility

To determine the reproducibility of the rctCap ELISA, six serum samples from farm pigs (three IFA-postitve samples and three IFA-negative samples) were selected for use in the reproducibility experiments. We ran four replicates of each serum sample on the same plate to assess the intra-assay (within-plate) reproducibility, and we ran five replicates of each sample on different plates to assess the inter-assay (between-run) reproducibility.

The results were used to calculate the mean S/P ratios and coefficients of variation (CV).

### IFA

Confluent PK15 cell monolayers, either untransfected or transfected with an infectious molecular clone of the P1 virus, in 96-well plates (Costar, Corning, NY, USA) were fixed in methanol-acetone for 15 min at −20 °C, and the plate was then washed three times with PBS (pH 7.2). Serum samples (50 μl) diluted 1:20 in PBST were added to the plates and incubated for 1 h at 37 °C. After three washes with PBST, fluorescein-conjugated anti-swine IgG antibody (KPL), diluted 1:100, was added and the samples were incubated for 30 min at 37 °C. Fluorescence was observed with an inverted fluorescence microscope (Olympus, Japan).

### Serum samples

A serological study was performed to estimate the seroprevalence of P1 in the swine population. Serum samples (*n* = 1135) were collected randomly from 11 herds with or without PMWS in the Jiangsu, Anhui, Shandong, and Zhejiang Provinces and Shanghai Municipality, China in 2008–2011. The origins of the serum samples were as follows: 86 and 70 serum samples were from farm A and farm B in Jiangsu Province, respectively; 72, 86, and 54 serum samples were from farm C, farm D, and farm E in Anhui Province, respectively; 42 and 106 serum samples were from farm F, and farm G in Shandong Province, respectively; 90 serum samples were from farm H in Shanghai Municipality; and 55, 69, and 405 serum samples were from farm I, farm J, and farm K in Zhejiang Province, respectively. The 405 serum samples from farm K in Zhejiang Province were obtained from different animal growth stages: suckling piglets (0–5 weeks, 140 sera), nursing pigs (8–13 weeks, 145 sera), fattening pigs (18–19 weeks, 60 sera), sows (45 sera), and breeding boar (15 sera).

## Results

### Cloning, expression and purification of the rctCap protein

**Fig. 2:**
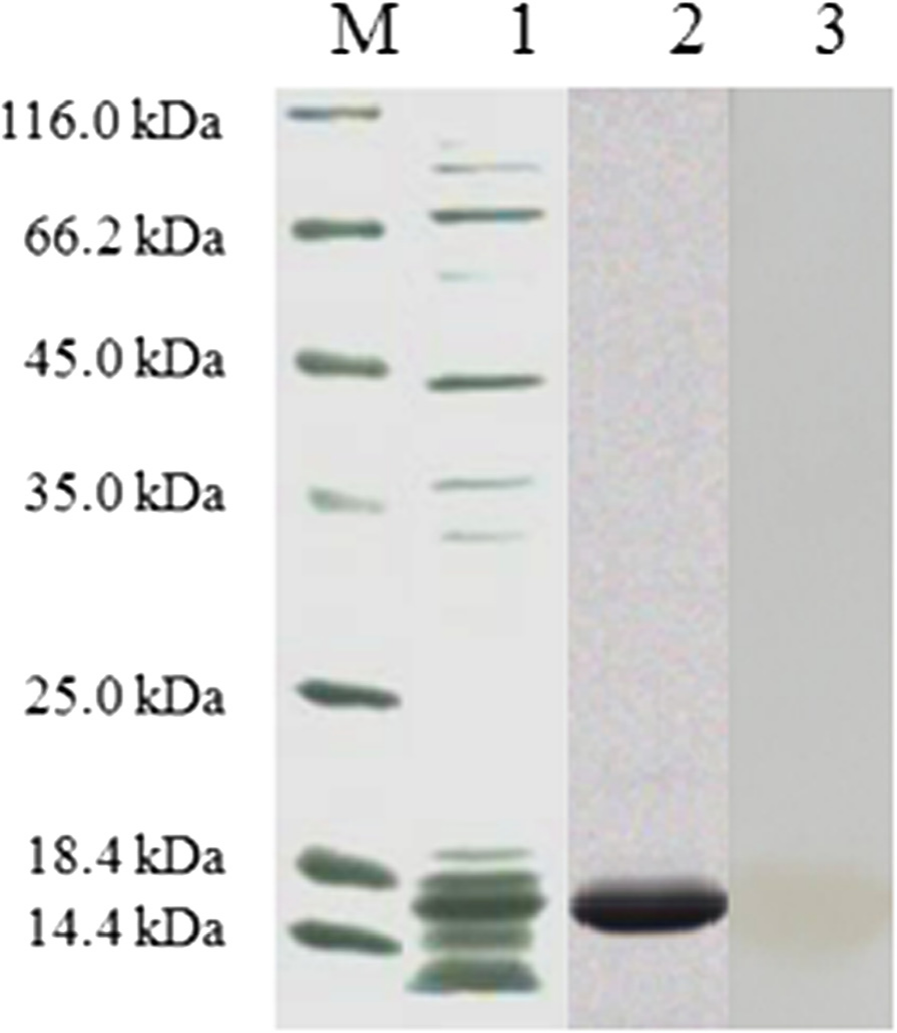
Identification of the rctCap protein with SDS-PAGE and western blotting. M: protein molecular weight marker; 1: IPTG-induced cell lysate; 2: rctCap protein purified with a Ni^2+^-NTA affinity chromatography column; 3: the purified rctCap protein reacted strongly with rabbit anti-P1 polyclonal antibody detected with HRP- conjugated goat anti-rabbit antibody as the secondary antibody

The truncated ORF1 was amplified with PCR and used with the appropriate primers to construct the recombinant truncated ORF1 fragment, which is composed of three repeated identical domains. The result was cloned into the pET-28a (+) vector, from which recombinant proteins were expressed as histidine (His) fusion proteins. After IPTG induction, the recombinant *E. coli* carrying the pET-28a-tri-ORF1 cassette expressed a 14.7-kDa protein, which corresponded to the expected size of the rctCap protein (containing an N-terminal peptide of six histidines). The recombinant protein was expressed as inclusion bodies in *E. coli*, and its size was confirmed with SDS-PAGE. *E.coli* carrying the recombinant plasmid were lysed with lysozyme (final concentration, 1 mg/ml) and sonication. The rctCap protein was purified from the lysate by using affinity chromatography. Most of the rctCap protein was eluted from a Ni^2+^-NTA affinity column. The purified protein was separated with SDS-PAGE and blotted onto a nitrocellulose membrane for immunodetection. The SDS-PAGE analysis of the eluted fractions revealed that the protein was purified as a single 15-kDa band. The reactivity of the rctCap protein to rabbit anti-P1 serum was observed with a western blotting assay (Fig. 2). These results confirm that this antigen can be used to detect specific antibodies directed against P1.

### Optimization of the rctCap ELISA

We used checkboard ELISAs to determine the optimal antigen concentration and the serum sample dilution, which were set at 2.62 μg/ml and 1:100, respectively, based on the criteria that under these conditions the OD_450_ value for positive serum was close to 1.0 and the P/N value was highest. After these conditions were met, other factors in the rctCap ELISA were optimized. We found that 1 % BSA was the best blocking solution for this assay., giving the highest P/N value, compared with the other tested blocking solutions The optimal dilution of the conjugate was 1:5000. When the optimal exposure time for the serum samples or detection antibody was investigated, incubation for 90 min or 45 min, respectively, was shown to be best.

### Reproducibility of the rctCap ELISA

The intra-assay and inter-assay reproducibility of the rctCap ELISA were determined by comparing the S/P ratios for each farm pig serum sample tested in triplicate. The intra-assay CVs of three serum samples that had been determined as P1 seropositive by IFA (IFA-positive) in the rctCap ELISA ranged from 1.5–3.3 %. The inter-assay CVs for IFA-positive serum samples in the rctCap ELISA ranged from 2.91–7.61 % (Table 2). The rctCap ELISA also showed good repeatability with three pig sera that had been determined as P1 seronegative by IFA (IFA-negative), as indicated by the low variability among the three replicates of identical samples. These data show that this assay is repeatable, yielding acceptably low levels of variation.

**Table 2:** Repeatability of the rctCap ELISA for IFA-positive serum samples of farm pig sera

Repeatability	Intra-assay			Inter-assay		
1	1.162	1.040	1.233	0.878	1.113	1.134
2	1.135	1.079	1.191	0.913	1.220	1.088
3	1.121	1.066	1.164	0.934	1.231	1.261
4	1.137	1.008	1.252	0.886	1.019	1.146
5	-	-	-	0.872	1.178	1.053
CV	1.50 %	3.00 %	3.30 %	2.91 %	7 .61 %	6.95 %

### Determination of the negative–positive cut-off

To set a negative**–**positive cut-off value for the rctCap ELISA, 900 presumed P1 seronegative farm swine sera collected from normal uninfected pigs were examined. The mean ± SD OD_450_ value for the normal pig sera in the rctCap ELISA was 0.21008 ± 0.04484, which gave a negative–positive cut-off S/*P* value of approximately 0.345 (mean + 3SD) for this assay. Therefore, serum samples with an OD_450_ > 0.345 in this assay were regarded as seropositive for P1.

### Specificity of the rctCap ELISA

The specificity of the rctCap ELISA was determined by testing the reactivity of 10 samples each of PPV, CSFV, PRV, PRRSV, or PCV2-positive sera, which came from the commercial ELISA kit. The results show that the mean OD_450_ values for the sera, described above were 0.193, 0.131, 0.185, 0.178, and 0.206, respectively. Therefore, based on our defined negative–positive cut-off value of OD_450_ > 0.345, none of these antisera was detected as P1 seropositive by the rctCap ELISA. These findings confirm that the rctCap antigen is specific for antibodies directed against P1.

### Validation of the rctCap ELISA

Of the 1135 swine serum samples described above, 450 randomly selected samples were assessed with both the rctCap ELISA and IFA. Of these, IFA found 162 samples as P1 seropositive and 288 samples as P1 seronegative, while evaluation with the rctCap ELISA at a cutoff value of 0.345 found 156 samples as P1 seropositive and 294 samples as P1 seronegative. When the samples were judged by both methods, 151 were considered P1 seropositive and 283 were considered P1 seronegative. Comparatively, five samples assessed as P1 seropositive by the rctCap ELISA were assessed as P1 seronegative by the IFA, and 11 samples determined as P1 seronegative by the rctCap ELISA were assessed as P1 seropositive by the IFA (Table 3). The sensitivity and specificity of the rctCap ELISA were 93.6 % and 98.3 %, respectively, based on the assumption that the IFA, considered the gold standard for P1 antibody detection, was 100 % accurate. The rate of accordbetween these two methods was 96.4 %.

**Table 3:** Comparison of the rctCAP ELISA with IFA for 450 farm pig serum samples

IIF results	ELISA results		
	Positive	Negative	Total
Positive	151	11	162
Negative	5	283	288
Total	156	294	450

### Results for farm pig serum samples assessed by the rctCap ELISA

The results for the 1135 farm pig serum samples tested with the rntCAP ELISA are presented in Table 4. Of these, 352 samples were positive and 783 samples were negative for anti-P1 antibodies. The seroprevalence of P1 in farm K in Zhejiang Province was 48.6 % for suckling piglets, 34.5 % for nursing pigs, 18.3 % for fattening pigs, 53.3 % for sows, and 20 % for breeding boars (Table 5).

**Table 4:** Prevalence of antibodies to P1 by ELISA in swine serum samples obtained from Jiangsu, Anhui, Shandong, Shanghai and Zhejiang

Farms	Jiangsu		Anhui			Shandong		Shanghai	Zhejiang		
	A	B	C	D	E	F	G	H	I	J	K
No. of sera tested	86	70	72	86	54	42	106	90	55	69	405
No. of sera positive	3	51	14	7	19	35	32	13	0	22	156
% sera positive	3.5	72.9	19.4	8.1	35.2	83.3	30.2	14.4	0	31.9	38.5

**Table 5:** Anti-P1 antibodies in different categories of pigs from Hangzhou pig farm K

	suckling piglets	nursing pigs	fattening pigs	sows	breeding boar
No. of sera tested	140	145	60	45	15
No. of sera positive	68	50	11	24	3
% sera positive	48.6	34.5	18.3	53.3	20

## Discussion

The increasing incidence of PMWS on pig farms has become a real threat to the global swine industry. Because P1 is associated with PMWS [16], detecting P1 in pig herds is essential for the control of this syndrome. Therefore P1-specific diagnostic tools are required to clarify the course of infection. To date, only PCR, IFA, and IPMA assay are used to detect P1 infection.

IFA is considered to be a sensitive method for detecting P1, but it requires virus-infected cells as well as experienced technicians to examine the stained plates, and it is laborious and time-consuming. Therefore, IFA is not suitable for large-scale surveys. The purpose of this study was to develop an indirect ELISA to detect antibodies specific for P1.

The N-terminal region of Cap contains one potential epitope shared by PCV2 and P1. Therefore, to avoid complications from antigenic cross-reactivity, we did not use the full-length Cap protein in this assay. The C-terminal 42 amino acids of Cap are unique to P1 and can be used to differentiate between antibodies directed against PCV2 and those directed against P1. However, because the conserved C-terminal region is very small, we constructed a plasmid containing three tandem repeats, each encoding the same 42 amino acids. Each 42-amino acid repeat was separated by 6 nt of a restriction enzyme recognition site, which created a two amino-acid linker between repeats. The plasmid was also constructed in frame with an N-terminal peptide of six histidines, which allowed the expressed capsid protein to be purified easily with chromatography. A strong specific signal was observed via western blotting, confirming that the truncated protein did not destroy the C-terminal epitope.

We compared the newly developed indirect ELISA for detecting P1-specific antibodies with an IFA. The data from the rctCap ELISA agreed quite well with the data from the IFA, with a relative sensitivity of 93.6 % and a specificity of 98.3 %.

There is high homology between the amino acid sequences of ORF2 of PCV2 and ORF1 of P1, especially in the N-terminal regions. Therefore, to determine the usefulness of the rctCap ELISA for P1, we had to evaluate its specificity against PCV2. Our results confirm that the rctCap ELISA does not react with antibodies directed against PCV2. Our data also demonstrate the specificity of this assay, which does not detect antibodies directed against PPV, CSFV, PRV, or PRRSV.

Epidemiological studies of P1 infections conducted on 11 farms in China showed that all of the herds were P1 seropositive, except for one, irrespective of the presence or absence of clinical signs of PMWS. A high P1 seroprevalence was observed in suckling piglets and sows (48.6 % and 53.3 %, respectively).

## Conclusions

The rctCap ELISA described in this study can be used for the detection of antibodies directed against P1, and this assay is highly sensitive, specific, and widely applicable to screening large numbers of serum samples. This report is the first to demonstrate different P1 serological profiles of pigs at different stages in herds with or without clinical signs of PMWS. However, further serological surveys are required to determine the dynamics of P1 infection in herds and the relationship of P1 to PMWS.
